# Loop Closure Detection Method Based on Similarity Differences between Image Blocks

**DOI:** 10.3390/s23208632

**Published:** 2023-10-22

**Authors:** Yizhe Huang, Bin Huang, Zhifu Zhang, Yuanyuan Shi, Yizhao Yuan, Jinfeng Sun

**Affiliations:** 1 Hubei Key Laboratory of Modern Manufacturing Quality Engineering, School of Mechanical Engineering, Hubei University of Technology, Wuhan 430068, China; yizhehuang@hbut.edu.cn (Y.H.); 102110149@hbut.edu.cn (B.H.); q1772698562@163.com (Y.Y.); 2 State Key Laboratory of Digital Manufacturing Equipment and Technology, Huazhong University of Science and Technology, Wuhan 430074, China; d201980200@hust.edu.cn; 3 Dongfeng Liuzhou Motor Co., Ltd., Liuzhou 545005, China; 4 School of Mechanical and Electrical Engineering, Hainan University, Haikou 570228, China; 996099@hainanu.edu.cn

**Keywords:** visual simultaneous localization and mapping, loop closure detection, similarity difference, convolutional neural network

## Abstract

Variations with respect to perspective, lighting, weather, and interference from dynamic objects may all have an impact on the accuracy of the entire system during autonomous positioning and during the navigation of mobile visual simultaneous localization and mapping (SLAM) robots. As it is an essential element of visual SLAM systems, loop closure detection plays a vital role in eradicating front-end-induced accumulated errors and guaranteeing the map’s general consistency. Presently, deep-learning-based loop closure detection techniques place more emphasis on enhancing the robustness of image descriptors while neglecting similarity calculations or the connections within the internal regions of the image. In response to this issue, this article proposes a loop closure detection method based on similarity differences between image blocks. Firstly, image descriptors are extracted using a lightweight convolutional neural network (CNN) model with effective loop closure detection. Subsequently, the image pairs with the greatest degree of similarity are evenly divided into blocks, and the level of similarity among the blocks is used to recalculate the degree of the overall similarity of the image pairs. The block similarity calculation module can effectively reduce the similarity of incorrect loop closure image pairs, which makes it easier to identify the correct loopback. Finally, the approach proposed in this article is compared with loop closure detection methods based on four distinct CNN models with a recall rate of 100% accuracy; said approach performs significantly superiorly. The application of the block similarity calculation module proposed in this article to the aforementioned four CNN models can increase the recall rate’s accuracy to 100%; this proves that the proposed method can successfully improve the loop closure detection effect, and the similarity calculation module in the algorithm has a certain degree of universality.

## 1. Introduction

Mobile robots are capable of determining their own motion trajectories in uncharted territory utilizing simultaneous localization and mapping (SLAM) [1,2], which enables the generation of maps of their surroundings. The application of SLAM technology is widespread in industries, including mobile robots, virtual reality [3,4], smart mobile homes, and autonomous driving [5]. Visual sensors are accessible and can capture detailed images; thus, visual SLAM with cameras has broad appeal [6]. However, variations with respect to perspective, lighting, weather, and interference from moving objects may all have a detrimental effect on the precision of the entire system when visual SLAM mobile robots perform autonomous positioning and navigation [7]. As a robot keeps moving, cumulative errors begin to occur when the robot uses images that a camera has taken and then comprehends the data within to obtain its own positioning and environmental observation data. Cumulative errors can only be eliminated by constraining the adjacent keyframes in the previous paragraph. By determining that the camera is returning to the same position, loop closure detection (LCD) can provide long-term keyframe constraints. When utilized in conjunction with the backend, it can generate globally consistent trajectories and maps and eliminate cumulative errors [8,9].

The current loop closure detection algorithm uses appearance information to address the issue of data association between images, and it is primarily based on the similarity matching method of the image and its data. The robot’s trajectory can vary in practical applications due to the interference of dynamic objects, which causes visual bias in the collected images [10], further resulting in inaccurate loop closure detection results. False negativity or perceptual bias are terms used to describe this phenomenon. The existence of localized similar scenes in various environments may also occur at the same time, leading to the accurate classification of a loop as a non-loop. False positives and perceptual confusion are terms used to describe this phenomenon. False negatives decrease the effectiveness of loop closure detection, while false positives cause map creation to fail and robot positioning to be lost [11,12]. As a result, the loop closure detection algorithm must increase loop closure recognition accuracy while reducing false positives. The bag-of-words (BoW) model, which represents the image using locally created features, is a frequently employed technique in the conventional loop closure detection algorithm [13]. The word vectors in this model are produced by clustering a large number of image feature vectors, but similarity comparisons are difficult due to the randomness of image collection and the limitations of clustering methods [14,15,16]. Some scholars have improved and proposed gridding place recognition (GPR) [17] and COVFast-LCD [18] methods on this basis. Deep learning has driven the development of computer vision and has achieved good results in areas such as image classification [19], object detection [20], instance segmentation [21], and object tracking [22]. The global description vector of an image can be successfully extracted using deep learning, offering a fresh approach to loop closure detection. An approach for loop closure detection based on convolutional neural networks (CNNs) was first proposed by Chen et al. in 2014 [23]. The Euclidean distance between the vectors used in this method to represent the similarity between images uses a pretraining network called Overfeel to extract image description vectors. The outcomes demonstrated that the loop closure detection effect outperforms FAB-MAP and SeqSLAM. The use of an autoencoder to extract the image description vector was suggested by Gao et al. [24]. An autoencoder is a type of unsupervised network model and does not need a lot of training using annotated images. This method exhibits good loop closure detection performance, as evidenced by the results, and uses a similarity matrix to represent the similarity between images. Merrill et al. [25] proposed a lightweight unsupervised deep neural network model, CALC, based on the autoencoder. The model trains the network model with the aim of extracting the global HoG descriptor of the image [26], and it randomly projects the input image to ensure that the output feature vectors have higher robustness relative to changes in perspective. The results show that the loop closure detection performance and real-time performance of this method are superior to the comparison algorithm. At the same time, there are NetVLAD and VGG-NetVLAD methods that combine the bag-of-words model with deep learning. NetVLAD combines the VLAD descriptor with CNN to propose a CNN architecture for weakly supervised location recognition. VGG-NetVLAD [27] combines NetVLAD with VGG16 to form a new algorithm.

Compared with the artificial features used in the traditional bag-of-words model, deep learning can extract more abundant image information [28] and is more robust in the case of light changes, viewpoint changes, etc. [29,30]. However, the accuracy of loop closure detection in deep learning depends on the performance of the deep learning network framework in extracting features and on the level of training. At present, deep learning based on loop closure detection algorithms is mostly focused on improving the robustness of image descriptors, ignoring the importance of similarity calculation and rarely paying attention to the connections between the internal regions of the image [31,32]. At present, although deep learning methods using local areal features may produce better results, these methods are complex in their calculations, require a large number of calculations, and are challenging in terms of ensuring that the extracted regional information is effective [33,34,35].

Therefore, we redesigned the loop closure detection algorithm in SLAM by combining MobileNet_v3 and block similarity calculation. The main work of this paper is as follows. (1) In comparing the precision–recall curves of existing excellent CNN models, the global descriptor of the image sequences is extracted using the pretrained lightweight neural network model MobileNet_v3 as the feature extractor in combination with the inverse residual structure in the network. (2) A principal component analysis (PCA) and whitening are used to improve the computational efficiency. (3) A block similarity calculation module is introduced to extract the local information of image block descriptors from the previously determined possible loop closure detection similarity pairs in fixed blocks and to re-judge the loop through a similarity calculation in order to improve the method’s loop detection accuracy. Finally, in order to verify the feasibility of the proposed method, a loop closure detection experiment is designed, and the results are analyzed. Experimental results show that the proposed method is effective and robust.

The structure of this paper is as follows: In Section 2, the overall framework of the designed algorithm is briefly introduced. Section 3 introduces the structure of the MobileNet_v3 network and the extraction and dimensionality reduction of image descriptors. Section 4 introduces the image block similarity calculation module in detail. In Section 5, the experimental results are discussed and analyzed, and in Section 6, the full text is summarized.

## 2. Method Framework

**Figure 1:**
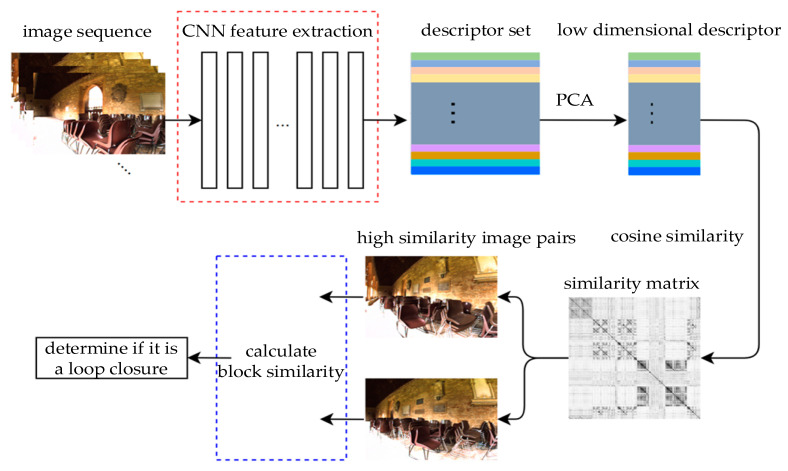
Loop closure detection method framework based on differences in the similarity of graphic blocks.

The method mainly consists of the extraction of image descriptors, the reduction of the dimensionality of image descriptors, and block similarity calculation. The overall framework of the method is shown in Figure 1.

Firstly, the pretrained CNN model is used to extract image descriptors and gather all the descriptors extracted from the image sequence. Then, the descriptors are subjected to a principal component analysis (PCA) and whitening to reduce some relevant dimensions, thereby reducing the subsequent computational complexity and preserving the main information. Finally, the cosine similarity of these descriptors is calculated, and a similarity matrix is generated. The similarity matrix is a symmetric matrix, each row of which can be regarded as a sequence of the current query image; each column can be regarded as a loop closure candidate. The query image is only compared with the image before the current time, and the image with the highest similarity to the query image can be found in this matrix. The two images with the most similarity are found, and the overall similarity is recalculated using the block similarity calculation module. The recalculated similarity is used to determine whether loop closure has occurred.

## 3. Image Descriptor

### 3.1. Image Descriptor Extraction

**Figure 2:**
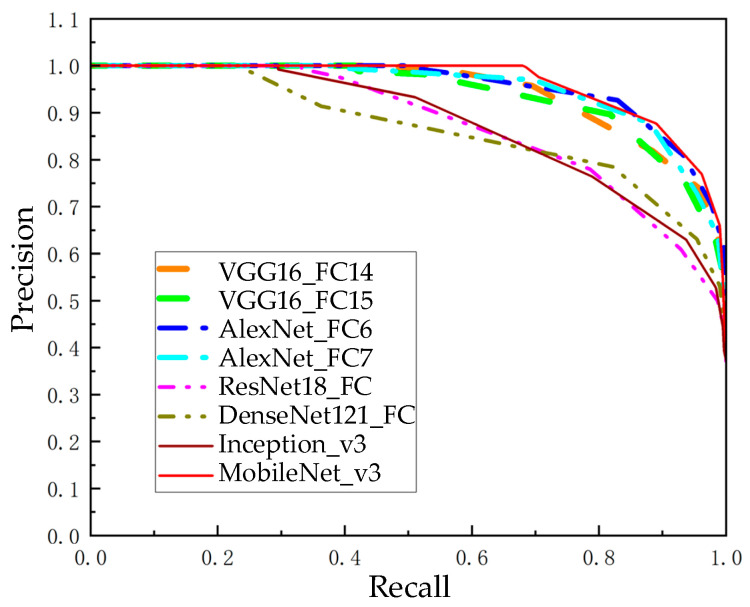
Precision–recall curves of distinct pretrained CNN models.

Directly calculating similarity from image data requires a significant amount of computation, and the results are frequently unreliable due to variables such as changing lighting, shifting viewpoints, and dynamic environments. An image descriptor is a vector used to represent an image, and representing the image as a vector is a necessary process for loop closure detection. Pretrained CNN models typically have good generalization performance while also reducing the time cost of retraining the network. Using a pretrained CNN to extract image features means richer image information can be used than with manually designed features. Visual Geometry Group 16 (VGG16) [36], AlexNet [37], Residual Network 18 (ResNet18) [38], MobileNet version 3 (MobileNet_v3), etc. [39], have shown good performance in practical applications such as image classification, image retrieval, image recognition, and other tasks. We performed loop closure detection on these CNN models using a public dataset from New College. Using the fully connected layers of these network models to extract image description vectors, the cosine similarity between the description vectors can be utilized to represent the similarity between images. The accuracy achieved with different recall rates is obtained by adjusting the similarity threshold, and these data are plotted into precision–recall curves. The precision–recall curves of different pretrained CNN models are shown in Figure 2.

**Figure 3:**
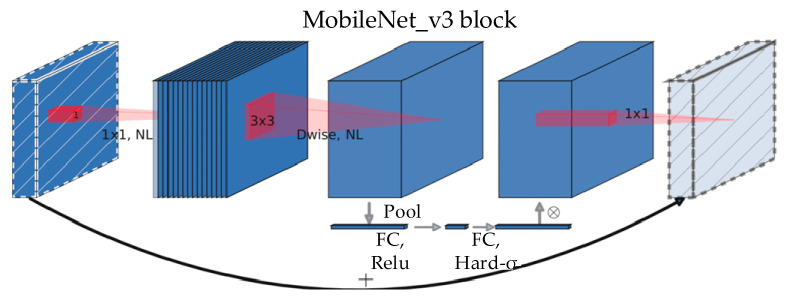
Diagram of Bneck’s structure.

Figure 2 shows that when compared with other pretrained CNN models, MobileNet_v3 has an outstanding recall rate and 100% accuracy. As a consequence, the pretrained CNN model chosen for image descriptor extraction is MobileNet_v3. Bneck is the primary processing method within the MobileNet_v3 network structure, and Figure 3 portrays its structure [39].

In order to meet the size requirements of the input image of the CNN model, the image size of the dataset should be adjusted to the input picture size of MobileNet_v3. To reduce the gradient value during the training, the model converges smoothly, and the image data are naturalized; the grayscale range of the image element is mapped from 0 to 255, in proportion, to between 0 and 1. After the naturalization process, the image is consistent with the original image, and the image’s information is unchanged.

At the same time, with standardized processing, the grayscale value of the image pixel point is mapped from −1 to 1, and decentralization is achieved; it is then easier for the image data to be subsequently generalized. Images are naturalized and standardized when training CNN models, so the input image is processed in the same way in order to achieve the extraction of characteristics. The calculation formula for image standardization is as follows:

(1) $$img\_std=\frac{x-\mu }{std}\;$$

In this case, img_std  represents the image matrix after standardized processing, with *x* representing the original image matrices, μ representing the average of the ImageNet dataset training images, and std representing the standard difference of the ImageNet dataset training images. μ and std are values of [0.485, 0.456, 0.406] and [0.229, 0.224, 0.225], respectively; these three components correspond to the three channels of the training image, and since these values are calculated from millions of images, they are directly credited when the input image processing is standardized.

The fully connected layer 1280-dimensional output vector of MobileNet_v3 is employed as the descriptor of the input image by the algorithm in order to verify the universality of the block similarity calculation method developed in the method. Other CNN models in the experiment also used the fully connected layer as the descriptor for the input image.

### 3.2. Image Descriptor Dimensionality Reduction

Due to the high number of sub-dimensions of the extracted image description, dimensionality reduction processing is needed to increase the speed of the subsequent cosine similarity calculation. Principal component analysis (PCA) can help vectors better represent images by reducing their dimensionality while also preserving the essential information in the vectors.

Suppose there are *m* images in the image sequence, the descriptor sub-dimension extracted from each image is *n*, and these image descriptors are combined together in rows to generate a generator matrix D, then D is expressed as

(2) $$D=\left[\begin{array}{cc} \begin{array}{cc} d_{11} & d_{12}\\ d_{21} & d_{22} \end{array} & \begin{array}{cc} \cdots & d_{1n}\\ \cdots & d_{2n} \end{array}\\ \begin{array}{cc} \vdots & \vdots \\ d_{m1} & d_{m2} \end{array} & \begin{array}{cc} \ddots & \vdots \\ \cdots & d_{mn} \end{array} \end{array}\right]$$

The specific calculation process of PCA is as follows.

(1) The mean is calculated for each column.

(3) $$\bar{d}=\left(\frac{1}{m}\sum_{i=1}^{m}d_{i1},\frac{1}{m}\sum_{i=1}^{m}d_{i2},\cdots ,\frac{1}{m}\sum_{i=1}^{m}d_{in}\right)=(\bar{d_{1}},\bar{d_{2}},\cdots ,\bar{d_{n}})$$

(2) The corresponding mean is subtracted from each column of D to obtain a matrix X centered around 0 for each column.

(4) $$X=D-{\left[\begin{array}{cc} \begin{array}{cc} \bar{d_{1}} & \bar{d_{2}}\\ \bar{d_{1}} & \bar{d_{2}} \end{array} & \begin{array}{cc} \cdots & \bar{d_{n}}\\ \cdots & \bar{d_{n}} \end{array}\\ \begin{array}{cc} \vdots & \vdots \\ \bar{d_{1}} & \bar{d_{2}} \end{array} & \begin{array}{cc} \ddots & \vdots \\ \cdots & \bar{d_{n}} \end{array} \end{array}\right]}_{m\times n}=\left[\begin{array}{cc} \begin{array}{cc} x_{1} & x_{2} \end{array} & \begin{array}{cc} \cdots & x_{n} \end{array} \end{array}\right]$$

(3) The covariance matrix X of matrix $X_{cov}$ is calculated.

(5) $$X_{cov}=\frac{1}{m}X^{T}X=\frac{1}{m}{\left[\begin{array}{cc} \begin{array}{cc} x_{1}\cdot x_{1} & x_{1}\cdot x_{2}\\ x_{2}\cdot x_{1} & x_{2}\cdot x_{2} \end{array} & \begin{array}{cc} \cdots & x_{1}\cdot x_{n}\\ \cdots & x_{2}\cdot x_{n} \end{array}\\ \begin{array}{cc} \vdots & \vdots \\ x_{n}\cdot x_{1} & x_{n}\cdot x_{2} \end{array} & \begin{array}{cc} \ddots & \vdots \\ \cdots & x_{n}\cdot x_{n} \end{array} \end{array}\right]}_{n\times n}$$

(4) Covariance matrix $X_{cov}$ undergoes singular value decomposition. As $X_{cov}$ is a symmetric matrix, its singular value decomposition form can be expressed as follows:
  (6) $$X_{cov}=U\Sigma U^{T}=\left[\begin{array}{cc} \begin{array}{cc} u_{1} & u_{2} \end{array} & \begin{array}{cc} \cdots & u_{n} \end{array} \end{array}\right]\cdot \left[\begin{array}{cc} \begin{array}{cc} \lambda_{1} & 0\\ 0 & \lambda_{2} \end{array} & \begin{array}{cc} \cdots & 0\\ \cdots & 0 \end{array}\\ \begin{array}{cc} \vdots & \vdots \\ 0 & 0 \end{array} & \begin{array}{cc} \ddots & \vdots \\ \cdots & \lambda_{n} \end{array} \end{array}\right]\cdot \left[\begin{array}{c} \begin{array}{c} {u_{1}}^{T}\\ {u_{2}}^{T} \end{array}\\ \begin{array}{c} \vdots \\ {u_{n}}^{T} \end{array} \end{array}\right]$$
  where $\lambda_{q}$, q∈ [1,2,⋯,n]. The non-zero part is the singular value of matrix X, arranged from largest to smallest, with the remaining values being 0. These singular values can be regarded as the contribution values of the dimension. $u_{p},p\in \;[1,2,\cdots ,n]$ is the vector obtained via the orthogonalization of the eigenvectors corresponding to singular values, and these vectors are arranged according to the corresponding singular values.
(5) The first k columns of matrix X and matrix U are multiplied for dimensionality reduction.
  (7) $$\begin{array}{l} D_{P}=XU_{k}={\left[\begin{array}{cc} \begin{array}{cc} x_{1} & x_{2} \end{array} & \begin{array}{cc} \cdots & x_{n} \end{array} \end{array}\right]}_{m\times n}\cdot {\left[\begin{array}{cc} \begin{array}{cc} u_{1} & u_{2} \end{array} & \begin{array}{cc} \cdots & u_{k} \end{array} \end{array}\right]}_{n\times k}\\ \;\;=\left[\begin{array}{cc} \begin{array}{cc} d_{11}^{\left(P\right)} & d_{12}^{\left(P\right)}\\ d_{21}^{\left(P\right)} & d_{22}^{\left(P\right)} \end{array} & \begin{array}{cc} \cdots & d_{1k}^{\left(P\right)}\\ \cdots & d_{2k}^{\left(P\right)} \end{array}\\ \begin{array}{cc} \vdots & \vdots \\ d_{m1}^{\left(P\right)} & d_{m2}^{\left(P\right)} \end{array} & \begin{array}{cc} \ddots & \vdots \\ \cdots & d_{mk}^{\left(P\right)} \end{array} \end{array}\right] \end{array}$$
  among them, $k\in N^{*}$ and k=min⁡(m,n). Through PCA dimension reduction, the dimensions of the image descriptor generator matrix D are reduced from *n* to *k*, and the main information is extracted. In order to reduce the correlation between various dimensions, whitening is usually performed after PCA dimensionality reduction, and the calculation process is as follows.
  (8) $$D_{W}=\left[\begin{array}{cc} \begin{array}{cc} \frac{d_{11}^{\left(P\right)}}{\sqrt{\lambda_{1}+\varepsilon }} & \frac{d_{12}^{\left(P\right)}}{\sqrt{\lambda_{2}+\varepsilon }}\\ \frac{d_{21}^{\left(P\right)}}{\sqrt{\lambda_{1}+\varepsilon }} & \frac{d_{22}^{\left(P\right)}}{\sqrt{\lambda_{2}+\varepsilon }} \end{array} & \begin{array}{cc} \cdots & \frac{d_{1k}^{\left(P\right)}}{\sqrt{\lambda_{k}+\varepsilon }}\\ \cdots & \frac{d_{2k}^{\left(P\right)}}{\sqrt{\lambda_{k}+\varepsilon }} \end{array}\\ \begin{array}{cc} \vdots & \vdots \\ \frac{d_{m1}^{\left(P\right)}}{\sqrt{\lambda_{1}+\varepsilon }} & \frac{d_{m2}^{\left(P\right)}}{\sqrt{\lambda_{2}+\varepsilon }} \end{array} & \begin{array}{cc} \ddots & \vdots \\ \cdots & \frac{d_{mk}^{\left(P\right)}}{\sqrt{\lambda_{k}+\varepsilon }} \end{array} \end{array}\right]=\left[\begin{array}{cc} \begin{array}{cc} d_{11}^{\left(W\right)} & d_{12}^{\left(W\right)}\\ d_{21}^{\left(W\right)} & d_{22}^{\left(W\right)} \end{array} & \begin{array}{cc} \cdots & d_{1k}^{\left(W\right)}\\ \cdots & d_{2k}^{\left(W\right)} \end{array}\\ \begin{array}{cc} \vdots & \vdots \\ d_{m1}^{\left(W\right)} & d_{m2}^{\left(W\right)} \end{array} & \begin{array}{cc} \ddots & \vdots \\ \cdots & d_{mk}^{\left(W\right)} \end{array} \end{array}\right]$$
  where $\varepsilon =10^{-4}$ is used to prevent situations where the denominator is 0.

After the aforementioned PCA dimensionality reduction and whitening processing, the image descriptor subcombination matrix D is transformed into a low-dimensional matrix $D_{W}$. Not only can it reduce the computational workload, but it can also retain the main information for subsequent similarity calculations.

## 4. Block Similarity Analysis

### 4.1. Image Pair Filtering

The reduced dimensionality image descriptors can better represent the image and facilitate calculation. The cosine similarity between these descriptors can be calculated directly to produce a similarity matrix. Through calculating the cosine value between two description vectors, which represents the angular distance between the two description vectors, the cosine similarity is obtained. The calculation equation is as follows:

(9) $$s(v_{A},v_{B})=\cos\left(\theta \right)=\frac{v_{A}\cdot v_{B}}{\left\|v_{A}\right\|\left\|v_{B}\right\|}$$

where term θ represents the angle between $v_{A}$ and $v_{B}$ in an *n*-dimensional vector space. As the cosine similarity increases and the vector angle decreases, the similarity between the images increases.

The images are read in chronological order, and the similarity between the images closest to the current query image and the query image is relatively high, so images near the query image are not detected. Of the remaining images, the image with the highest similarity to the query image is used for subsequent block similarity calculation.

**Figure 4:**
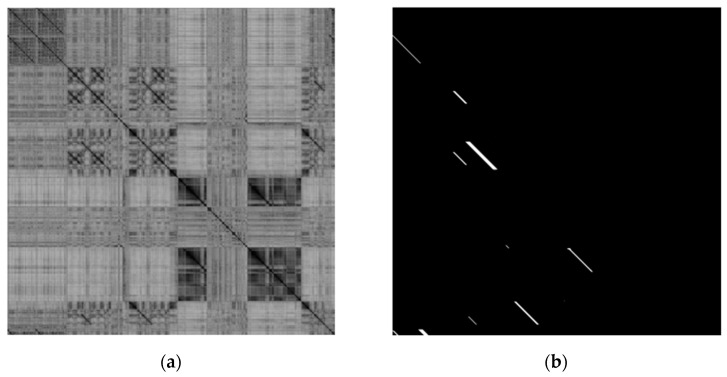
Similarity matrix and loop closure matrix of the New College dataset: (**a**) similarity matrix in the MobileNet_v3 model; (**b**) loop closure matrix.

Figure 4 shows the similarity matrix calculated using the dataset from New College and the MobileNet_v3 model and shows the true loop closure matrix of the dataset itself.

The similarity matrix is used to measure the similarity between the query image and the loop closure candidate image. This matrix is a symmetric matrix. The value at (*i*, *j*) represents the similarity between the *i*-th image and the *j*-th image in the dataset. Therefore, the value on the diagonal is 1. The darker the color in Figure 4, the higher the similarity. Only the lower triangular matrix area of the loop closure matrix has values, and the white area indicates that there is loop closure. Figure 4 shows that the regions with higher similarity in the similarity matrix and calculated directly using cosine similarity have some overlap with the real loop closure regions in the loop closure matrix. However, there are still many false positives among them. The main reason for this is that the method of directly using the global descriptor of the image to calculate similarity is not sensitive enough to some locally changing images. Therefore, in addition to the global information of the image to calculate similarity, the local information of the image can be utilized. However, it often cannot be determined whether changes in the local area of the image are caused by correct looping in a dynamic environment or by incorrect looping due to local differences, which can easily lead to false negatives. Therefore, it is also necessary to utilize the interrelationships between the local regions of the image to connect the local information of the image with the overall image.

### 4.2. Blocking Similarity

**Figure 5:**
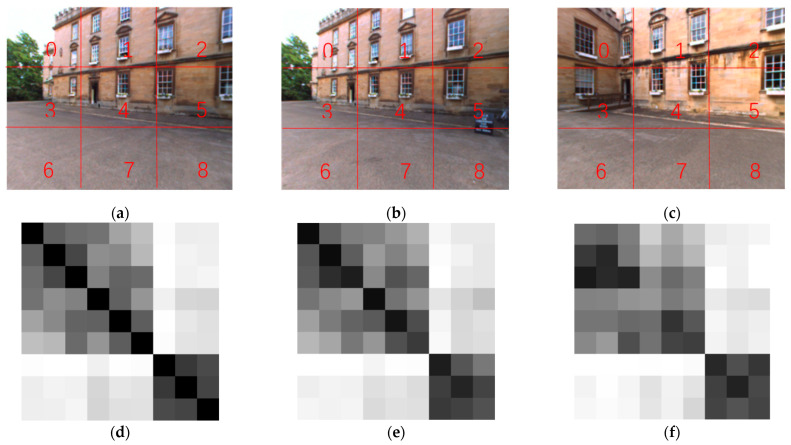
(**a**) img1; (**b**) img2; (**c**) img3; (**d**) the similarity matrix between img1 and img1 image blocks; (**e**) the similarity matrix between img1 and img2 image blocks; (**f**) the similarity matrix between img1 and img3 image blocks.

In order to utilize the local information in the images and obtain some images from the New College dataset for experiments, the given query image and loop closure candidate image are evenly divided into nine image blocks. Then, each small block of the image is input into MobileNet_v3, and nine description vectors are obtained for each image. The cosine similarity is used to calculate the similarity between the query image and loop closure candidate image, as well as between the query image and its own image block. A matrix similarity is then built based on image blocks, as shown in Figure 5.

In Figure 5, subgraphs (a) and (b) are correct loops, while subgraphs (a) and (c) are incorrect loop closures. Subgraph (d) is the matrix similarity SM1 between each image block of subgraph (a) and all image blocks of subgraph (a). Subgraph (e) is the matrix similarity SM2 between each image block of subgraph (a) and all image blocks of subgraph (b). Subgraph (f) is the matrix similarity SM3 between each image block of subgraph (a) and all image blocks of subgraph (c).

SM1, SM2, and SM3 are all 9 × 9 matrices that are normalized. The value located at (*i*, *j*) represents the similarity between the *i*-th and *j*-th image blocks in the image. It can be seen from subgraph (e) and subgraph (f) that the elements on the main diagonal of the matrix similarity of the correct loop closure image pair are darker and more similar than the elements on the matrix similarity of the wrong loop closure image pair, which indicates that the overall similarity between the loop closure image pairs can be expressed by the elements of the main diagonal of the matrix similarity, to a certain extent. However, in order to avoid the false negative results caused by direct calculation, it is necessary to further use the connection between image blocks, thereby connecting the local information of the image with the overall information. Comparing subgraphs (d) with (e) and (f), it can be found that the correct loop closure image is more similar to the matrix similarity as a whole, while the error loop closure image is more different from the matrix similarity as a whole. Therefore, the similarity of image pairs is recalculated based on the similarity difference between the image blocks mentioned above, further distinguishing between correct and incorrect loops.

**Figure 6:**
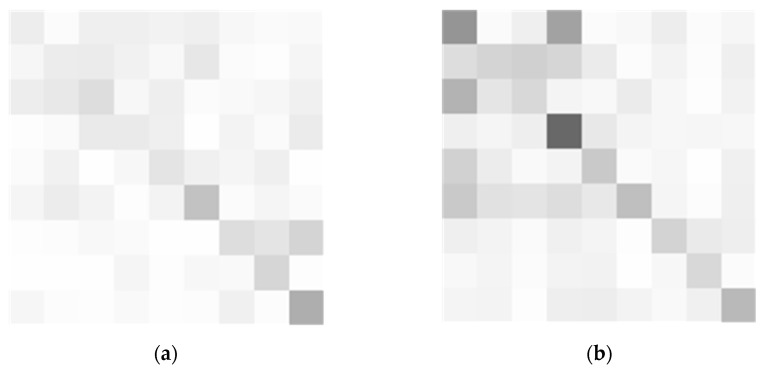
(**a**) Subtracting SM1 and SM2 to obtain the similarity difference matrix SM_d1; (**b**) subtracting SM1 and SM3 to obtain the similarity difference matrix SM_d2.

To more intuitively represent the difference between the correct loop closure image pair and the incorrect loop closure image pair, the matrices SM1 and SM2 are subtracted and taken as absolute values, and the matrices SM1 and SM3 are subtracted and taken as absolute values. The calculation process is as follows, and the results are shown in Figure 6.

(10) $$d_{i}=\sum_{j=0,j\neq i}^{8}\left|{SM}_{ij}-{SM}_{ij}^{\prime }\right|,i\in \left\{0,1,2,\ldots ,8\right\}$$

where $d_{i}$ represents the similarity difference between the query image and the *i*-th image block of the loop closure candidate image.

In Figure 6, subgraph (a) represents the similarity difference matrix SM_d1, obtained by subtracting the absolute values of SM1 and SM2; subgraph (b) represents the similarity difference matrix SM_d2, obtained by subtracting the absolute values of SM1 and SM3.

SM_ d1 and SM_ d2 both are 9 × 9 matrices located at (*i*, *j*); they represent the difference in similarity between the *i*-th and *j*-th image blocks in the image pair. The lighter the color, the smaller the similarity difference, while the darker the color, the greater the similarity difference. The correct loop closure image has a lighter color representing the similarity difference matrix compared with the incorrect loop closure image, indicating that the loop closure image has a smaller overall difference.

Each line of SM_d1 except the elements on the main diagonal is added to obtain the similarity difference value of nine image blocks: K_1 = [0.16, 0.17, 0.20, 0.16, 0.13, 0.14, 0.17, 0.05, 0.08]. Each line of SM_d2 except the elements on the main diagonal is added to obtain the similarity difference value of nine image blocks: K_2 = [0.30, 0.34, 0.32, 0.19, 0.22, 0.39, 0.19, 0.11, 0.18]. Each element in K_1 and K_2 represents the overall similarity difference between the corresponding image blocks in the correct looping image pair and the incorrect looping image pair; the smaller the value, the smaller the difference. Through more intuitive data comparison, it can be found that individual values in K_2 are not significantly different from those in K_1, but overall, K_2 has a larger value than K_1. Therefore, the similarity difference value can be used to recalculate the similarity between image pairs, thereby reducing the similarity of error loops to a greater extent.

In observing subgraph (d), subgraph (e), and subgraph (f) in Figure 5, it can be found that the elements on the main diagonal, that is, the similarity between image blocks in the same position, can represent the overall similarity of the image. However, this will neglect the connection between the local areas of the image and the overall image, causing the overall similarity to be significantly affected by local area similarity. Particularly in some cases of local environmental changes, significant changes in similarity are easily caused, meaning this method is not suitable for dynamic environments. Therefore, the matrix similarity SM main diagonal elements are weighted, and the weight distribution of the algorithm is as follows:

(11) $$\lambda_{i}=1-\left(1+0.1\;k\right)\;d_{i},\;k\in \left\{-10,-9,\ldots ,0,\ldots ,9\;,10\right\}$$

where the term $\lambda_{i}$ represents the similarity weight between the query image and the *i*-th image block of the loop closure candidate image. The term *k* is an adjustment parameter used to indicate the degree to which similarity differences ($d_{i}$) affect weights ($\lambda_{i}$). *k* = −10 indicates that $d_{i}$ has no effect on $\lambda_{i}$, while the larger the value of *k*, the greater the impact of $d_{i}$ on $\lambda_{i}$. From Equation (11), the weight ($\lambda_{i}$) is a number less than 1; the smaller the similarity difference ($d_{i}$), the closer n is to 1. The larger the similarity difference ($d_{i}$), the smaller $\lambda_{i}$ is.

The overall similarity between the query image and the loop closure matching image is denoted as *Sim*, and the similarity between the corresponding image blocks after the query image and the loop closure candidate image are segmented is denoted as ${Sim\_a}_{i},\;i\in \left\{0,\;1,\;2,\ldots ,8\right\}$. The similarity after recalculation is represented as follows:

(12) $$S=\frac{Sim\cdot \sum_{i=0}^{8}\lambda_{i}\cdot {Sim\_a}_{i}}{\sum_{i=0}^{8}{Sim\_a}_{i}}$$

From Equation (12), the closer $\lambda_{i}$ is to 1, the closer the recalculated similarity is to the overall image similarity of *Sim*. The smaller $\lambda_{i}$ is, the smaller the similarity after recalculation.

### 4.3. Numerical Calculation

**Figure 7:**
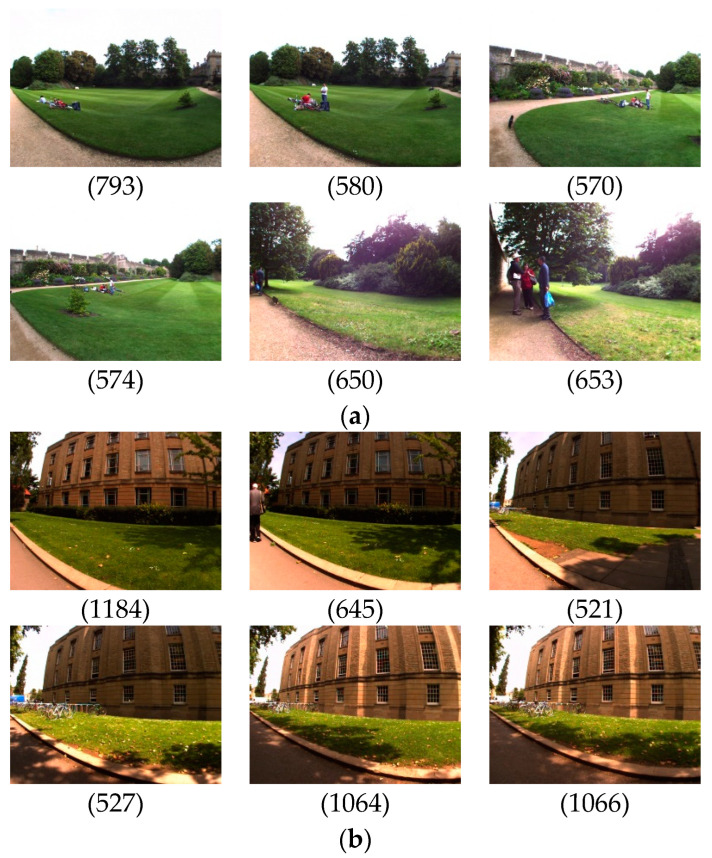
Dataset loop closure comparison between (**a**) New College and (**b**) City Center datasets.

To visualize the calculations, a set of images are separately extracted from the New College and City Center datasets. Take the New College dataset, for example, which includes an image to be queried, serial number 793; a correct loop closure image, serial number 580; and four images with similarities to images to be interrogated, with serial numbers 570, 574, 650, and 653, respectively. The City Center dataset images are extracted using the same method, as shown in Figure 7.

The adjustment parameter k=−7 is used to calculate the likeness of the image before and after the image partition with the image to be queried, as well as the similarity difference for each image block, as shown in Table 1 and Table 2.

From the comparison data, it can be observed that images with a higher similarity but non-loop closure have higher differential values of similarity compared to the loop closure image pair, and after the calculation of the similarity of the block, similarity decreases more, which is more conducive to judging the correct loop closure.

## 5. Results and Discussion

### 5.1. Experimental Environment and Datasets

**Figure 8:**
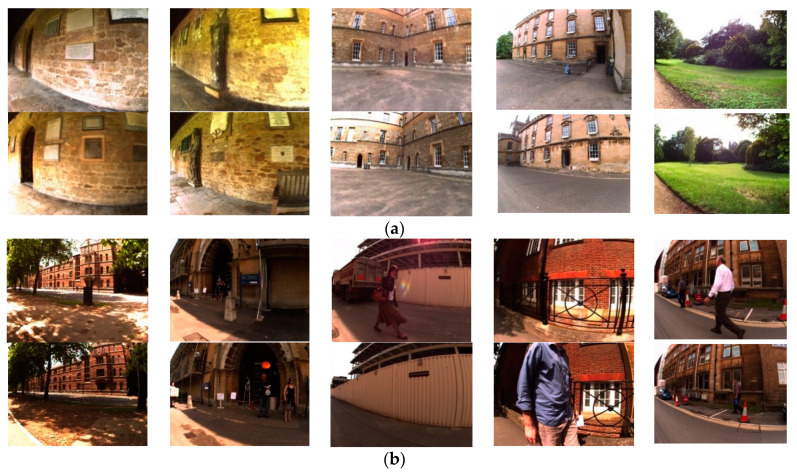
Partial dataset images from the (**a**) New College dataset and (**b**) City Center dataset.

The experiment used two publicly available datasets, namely, the New College and City Center datasets. New College and City Center are datasets provided by the Mobile Robotics Group of the University of Oxford [40]. During the robot’s image acquisition process, images are collected approximately every 1.5 m and are commonly used for loop closure detection evaluation testing. Images of the dataset are shown in Figure 8. The detailed parameters of the dataset are shown in Table 3. Both datasets are composed of binocular images, and only the left images are utilized in this experiment.

### 5.2. Discussion of Experimental Results

**Figure 9:**
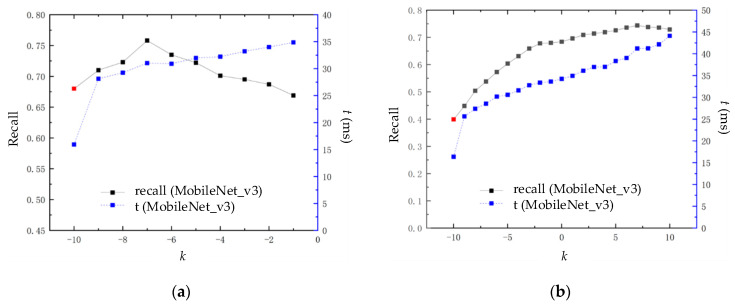
Effect of adjusting parameter *k* on the loop closure detection performance of the algorithm: (**a**) loop closure detection performance using the New College dataset; (**b**) loop closure detection performance using the City Center dataset.

Within the block similarity calculation module, an adjustment parameter *k* is added. The algorithm can adjust for various environments via altering the value of *k*. As a result, the parameter *k* impacts the extent to which the algorithm performs in terms of time and loop closure detection. The effectiveness of the loop closure detection is expressed through accuracy and recall. Accuracy is defined as the ratio of the correct loop closure detected (true positive (TP)) to all loop closure, as determined by the algorithm (true positive (TP) and false positive (FP)). The recall rate is defined as the ratio of detected correct loop closure (true positive (TP)) to all loop closure (true positive (TP) and false negative (FN)) in the dataset. Due to the occurrence of false positives leading to errors in backend optimization algorithms, the loop closure detection effect is represented by a recall (%) with 100% accuracy. The time performance is represented by the average query time *t* (ms) of each image in the algorithm. The experimental results are shown in Figure 9.

In Figure 9, subfigure(a) represents the experimental data from the New College dataset, and subfigure (b) represents the experimental data from the City Center dataset. The red dot at *k* = −10 indicates the recall rate of the loop closure detection algorithm based on the MobileNet_v3 CNN model at 100% accuracy, without applying the block similarity calculation module. The experimental results show that as parameter *k* increases, the loop closure detection performance of this algorithm first increases and then decreases. Within a certain range, the recall rate at 100% accuracy is higher than the red dot; outside of this range, it will be lower than this point. This is mainly because as parameter *k* increases, the similarity of error loop closure image pairs with similar appearances gradually increases compared to many correct loop closure image pairs with less similar appearances, resulting in a decrease in recall rate at 100% accuracy.

The average detection time of the method consists of three parts: image descriptor extraction, image descriptor reduction, and block similarity calculation. On the New College dataset, this algorithm performs best in loop closure detection when *k* = −7. At this point, the recall rate at 100% accuracy is 0.758, and the average detection time is 31.02 ms. On the City Center dataset, this algorithm performs best in loop closure detection when k = 7. At this point, the recall rate at 100% accuracy is 0.744, and the average detection time is 41.21 ms. The improvement in the loop closure detection effect is accompanied by a decrease in time efficiency, which is mainly reflected in the calculation process of block similarity. This is due to the need to input image blocks into the CNN model again to recalculate similarity, which increases time consumption. However, the average calculation time still meets the real-time requirements of loop closure detection, and greater time efficiency can be achieved by taking a smaller *k* value.

**Figure 10:**
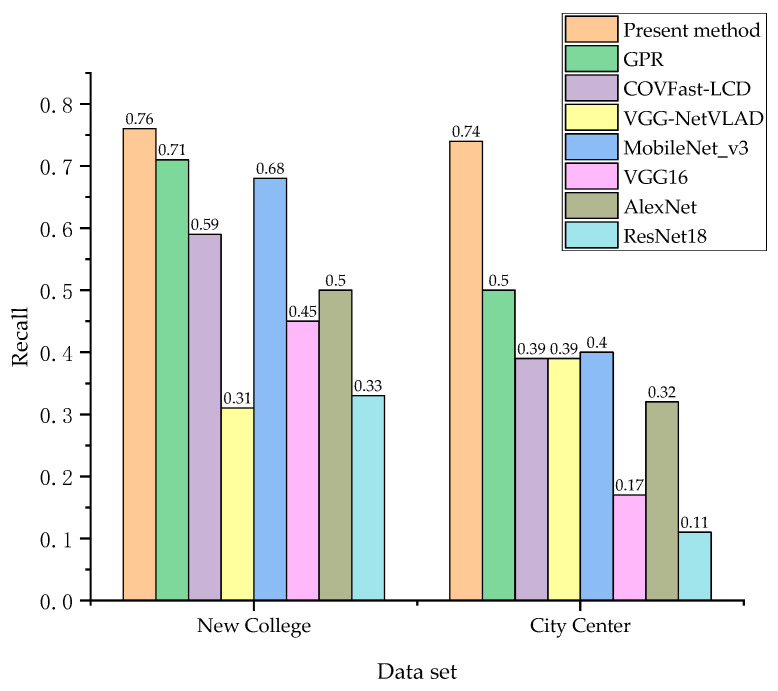
Loop closure detection performance of the algorithm on two datasets: the New College dataset and City Center dataset.

We undertook a comparison of the present method with the loop closure detection algorithms of four CNN models: GPR, COVFast-LCD, VGG-NetVLAD, MobileNet_v3, VGG16, AlexNet, and ResNet18; the recall rates at 100% accuracy on two datasets are shown in Figure 10.

As depicted in Figure 10, compared with the loop closure detection algorithms based on four CNN models—MobileNet_v3, VGG16, AlexNet, and ResNet18—the proposed present method has improved recall rates at 100% accuracy. The results show that this method can effectively improve the loop closure detection effect. Compared to the New College dataset, this method demonstrated a significant improvement in performance on the City Center dataset. There are many dynamic environments in the City Center dataset, indicating that the present method is more suitable for such scenarios.

**Figure 11:**
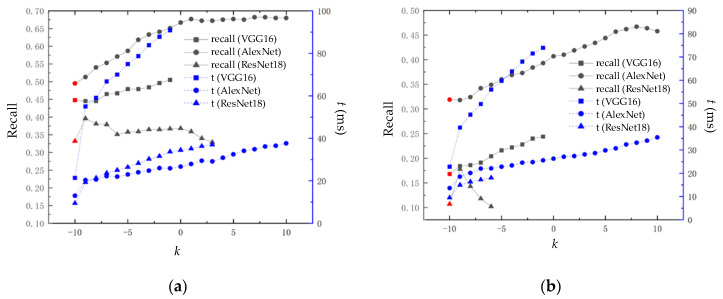
Loop closure back detection effect applied to three CNN models: VGG16, AlexNet, and ResNet8: (**a**) loop closure detection performance on the New College dataset; (**b**) loop closure detection performance on the City Center dataset.

The block similarity calculation module proposed in the present method is applied to three CNN models: VGG16, AlexNet, and ResNet18. The experimental results are shown in Figure 11.

In Figure 11, the red dot at *k* = −10 represents the recall rate at 100% accuracy of the loop closure detection algorithm based on the CNN model without the application of the block similarity calculation module. We found that the experimental results show a similar trend to the experimental results in Figure 9; the VGG16, AlexNet, and ResNet8 CNN models have improved recall rates at 100% accuracy after the application of the block similarity calculation module. This indicates that the block similarity calculation module can effectively improve the loop closure detection effect, reflecting the universality of the block similarity calculation module. It is worth noting that VGG16, due to the long time needed to extract image descriptors, increases rapidly with the increase in *k*, resulting in difficulties in real-time performance.

## 6. Conclusions

This article presents the use of the MobileNet_v3 neural network model to extract image descriptors and presents a block similarity calculation module used to reduce the similarity of error loop closure image pairs. We successfully improved the recall rate of the visual SLAM loop closure detection method at 100% accuracy, while meeting real-time requirements. Regarding the New College and City Center datasets, this method increased the recall rate at 100% accuracy by 8%, 31%, 26%, and 43% and by 34%, 57%, 42%, and 63%, respectively, compared to four CNN models based on MobileNet_v3, VGG16, AlexNet, and ResNet18. The three CNN models VGG16, AlexNet, and ResNet18 show improved recall rates at 100% accuracy after the block similarity calculation module is applied, demonstrating the method’s universality.

## Figures and Tables

**Table 1:** New College dataset calculation results.

		Image Similarity to 793 to Be Queried		Block Similarity Difference Value								
				Block								
	Image Serial Number	Before Block Calculation	After Block Calculation	1	2	3	4	5	6	7	8	9
Images to be queried	793	1	1	0	0	0	0	0	0	0	0	0
Loop closure	580	0.87	0.81	0.21	0.25	0.25	0.19	0.19	0.27	0.16	0.17	0.26
Non-loop closure	570	0.71	0.42	0.51	0.82	0.91	0.41	0.61	0.74	0.34	0.34	0.73
	574	0.73	0.41	0.62	0.73	0.81	0.44	0.62	0.63	0.49	0.36	0.66
	650	0.74	0.45	0.55	0.62	0.63	0.42	0.58	0.31	0.49	0.39	0.48
	653	0.59	0.35	0.63	0.74	0.52	0.43	0.80	0.54	0.49	0.45	0.60

**Table 2:** City Center dataset calculation results.

		Image Similarity to 1184 to Be Queried		Block Similarity Difference Value								
				Block								
	Image Serial Number	Before Block Calculation	After Block Calculation	1	2	3	4	5	6	7	8	9
Images to be queried	1184	1	1	0	0	0	0	0	0	0	0	0
Loop closure	645	0.91	0.83	0.20	0.27	0.18	0.17	0.16	0.19	0.13	0.20	0.25
Non-loop closure	521	0.73	0.53	0.21	0.36	0.41	0.55	0.61	0.55	0.38	0.50	0.51
	527	0.72	0.52	0.31	0.41	0.33	0.59	0.83	0.77	0.37	0.46	0.48
	1064	0.73	0.55	0.26	0.23	0.37	0.48	0.65	0.54	0.33	0.48	0.55
	1066	0.71	0.49	0.26	0.34	0.38	0.57	0.78	0.73	0.39	0.49	0.58

**Table 3:** Dataset information.

Dataset	New College	City Center
Total length (m)	2260	2025
Revisit length (m)	1570	801
Number of images	1073	1237
Resolution (px × px)	640 × 480	640 × 480

## Data Availability

Data are available on request.
